# Epidemiologic and clinical features of patients with COVID-19 in Brazil

**DOI:** 10.31744/einstein_journal/2020AO6022

**Published:** 2020-08-12

**Authors:** Vanessa Damazio Teich, Sidney Klajner, Felipe Augusto Santiago de Almeida, Anna Carolina Batista Dantas, Claudia Regina Laselva, Mariana Galvani Torritesi, Tatiane Ramos Canero, Otávio Berwanger, Luiz Vicente Rizzo, Eduardo Pontes Reis, Miguel Cendoroglo

**Affiliations:** 1 Hospital Israelita Albert Einstein São PauloSP Brazil Hospital Israelita Albert Einstein , São Paulo , SP , Brazil .

**Keywords:** Communicable diseases, Lung diseases/epidemiology, SARS-CoV-2, COVID-19, Coronavirus infections, Epidemiology

## Abstract

**Objective:**

This study describes epidemiological and clinical features of patients with confirmed infection by SARS-CoV-2 diagnosed and treated at *Hospital Israelita Albert Einstein* , which admitted the first patients with this condition in Brazil.

**Methods:**

In this retrospective, single-center study, we included all laboratory confirmed COVID-19 cases at *Hospital Israelita Albert Einstein* , São Paulo, Brazil, from February until March 2020. Demographic, clinical, laboratory and radiological data were analyzed.

**Results:**

A total of 510 patients with a confirmed diagnosis of COVID-19 were included in this study. Most patients were male (56.9%) with a mean age of 40 years. A history of a close contact with a positive/suspected case was reported by 61.1% of patients and 34.4% had a history of recent international travel. The most common symptoms upon presentation were fever (67.5%), nasal congestion (42.4%), cough (41.6%) and myalgia/arthralgia (36.3%). Chest computed tomography was performed in 78 (15.3%) patients, and 93.6% of those showed abnormal results. Hospitalization was required for 72 (14%) patients and 20 (27.8%) were admitted to the Intensive Care Unit. Regarding clinical treatment, the most often used medicines were intravenous antibiotics (84.7%), chloroquine (45.8%) and oseltamivir (31.9%). Invasive mechanical ventilation was required by 65% of Intensive Care Unit patients. The mean length of stay was 9 days for all patients (22 and 7 days for patients requiring or not intensive care, respectively). Only one patient (1.38%) died during follow-up.

**Conclusion:**

These results may be relevant for Brazil and other countries with similar characteristics, which are starting to deal with this pandemic.

## INTRODUCTION

Since December 2019, several cases of pneumonia of unknown origin have been reported in Wuhan, China. ^( 1 )^ The pathogen was further identified as a novel RNA coronavirus, currently named as severe acute respiratory syndrome coronavirus 2 (SARS-CoV-2). ^( 2 )^ Huang et al., reported the first cases in China, with a common clinical presentation of fever, cough, myalgia, fatigue and dyspnea, with organ dysfunction ( *e.g* ., acute respiratory distress syndrome – ARDS, shock, acute cardiac and kidney injuries) and death, in severe cases. ^( 3 )^

Afterwards, in January 2020, the World Health Organization (WHO) declared the outbreak a Public Health Emergency of International Concern (PHEIC), and next, in March 2020, it was characterized as a pandemic. ^( 4 )^ As of April 7, 2020, a total of 1,429,437 cases had been reported in 184 countries and regions across all five continents, with 82,074 deaths worldwide. ^( 5 )^ More recently, the Chinese Center for Disease Control and Prevention published data on 72,314 patients, with 44,672 (62%) classified as confirmed cases of coronavirus disease 2019 (COVID-19). Most patients were aged 30 to 79 years (87%), with mild clinical presentation (81%; *i.e* ., non-pneumonia and mild pneumonia) and overall case-fatality rate of 2.3% (increased in elderly population, with case-fatality rate of 14.8% in those aged 80 years and older). ^( 6 )^

On February 26, 2020, the first Brazilian patient had a confirmed diagnosis of COVID-19 at *Hospital Israelita Albert Einstein* (HIAE). *Hospital Israelita Albert Einstein* is a philanthropic hospital in the city of São Paulo (SP), Brazil, with twelve health care units, including a quaternary hospital with 592 beds, and four outpatient emergency care units. By the end of this study, on March 25, 2020, of 2,433 patients with confirmed COVID-19 in Brazil, 32% (769) had been diagnosed at HIAE.

Given the rapid spread of the COVID-19, clinical and epidemiological data of several countries are being published on a daily basis. ^( 7 - 9 )^ However, no studies have been reported to date presenting the characteristics of COVID-19 patients diagnosed in Brazil.

## OBJECTIVE

To describe epidemiological and clinical features of patients with confirmed infection by SARS-CoV-2, diagnosed and treated at *Hospital Israelita Albert Einstein* , which admitted the first patients with this condition in Brazil.

## METHODS

### Study design and oversight

This was a retrospective, observational, single-center study, which included all consecutive patients with a confirmed diagnosis of COVID-19, at HIAE, between February 26, 2020 and March 25, 2020. The study was supported by an internal grant from HIAE and designed by the investigators. The study was approved by the Research Ethics Committee of the organization, protocol number 3.921.190, CAAE: 30047620.3.0000.0071, and the National Commission for Research Ethics.

### Patients

The diagnosis of the COVID-19 disease was performed according to the WHO interim guidance. ^( 10 )^ A confirmed case of COVID-19 was defined as a positive result of real-time reverse transcriptase polymerase chain reaction (RT-PCR) assay of nasal and pharyngeal swab specimens. ^( 11 )^ All cases included in the current analysis had laboratory confirmation.

### Data sources

The data were obtained from patients’ electronic medical records (EMR), including inpatients and outpatients with laboratory-confirmed COVID-19. Data collected included demographic, clinical, laboratorial and radiological information, and was anonymized so that patients could not be identified.

Demographic characteristics included age, sex, tobacco smoking, weight and body mass index (BMI). Clinical information included medical, travel and exposure history, signs, symptoms, underlying comorbidities, continuous medication use and treatment measures ( *i.e* ., antiviral therapy, steroid therapy, respiratory support and kidney replacement therapy). Laboratory assessment consisted of complete blood count, assessment of renal and liver function, and measurements of electrolytes, D-dimer, procalcitonin, lactate dehydrogenase, C-reactive protein, and creatine kinase. Radiologic abnormality was defined based on the medical report documented in the EMR. Disease duration from onset of symptoms, hospital and Intensive Care Unit (ICU) length of stay (LOS) were also documented.

### Statistical analysis

Continuous variables were expressed as means with standard deviations, medians, minimum and maximum values. Categorical variables were summarized as counts and percentages. No imputation was made for missing data. All statistics are deemed to be descriptive only, considering that the cohort of patients in our study was not derived from random selection. All analyses were performed using Microsoft Excel 2013.

## RESULTS

### Demographic and clinical characteristics

Between February 26 and March 25, 2020, a total of 769 patients were diagnosed with COVID-19 at HIAE. This study included 510 (66%) patients, for whom data regarding demographics, clinical symptoms, laboratory and imaging findings were available in the EMR. The remaining 259 patients had only used the hospital laboratory facilities, and were followed-up by physicians not working in our service network.

Patients’ demographic and clinical characteristics are shown in table 1 . A total of 34.4% had a recent international travel history and 5.7% had been at the same marriage celebration in Bahia, a state in the Northeast region of Brazil; 184 (61.1%) patients had a history of close contact either with a positive or suspected case of COVID-19. Most patients were male (56.9%) and the mean age was 40 years. Only 0.6% of patients were younger than 11 years old and 6.5% were older than 65 years.

**Table 1:** Clinical and epidemiological characteristics

Characteristic	Total patients (n=510)	Total patients	
		Non-hospitalized patients (n=438)	Hospitalized patients (n=72)
Age, years			
Mean±SD	39.9±13.6	38.6±12.5	51.8±15.32
Median	38.0	37.0	52.0
Minimum-Maximum	2-92	2-84	5-92
Number of patients	510	438	72
Age distribution, years			
0-11	3/510 (0.6)	2/438 (0.5)	1/72 (1.4)
12-49	400/510 (78.4)	369/438 (84.2)	31/72 (43.1)
50-64	74/510 (14.5)	49/438 (11.2)	25/72 (34.7)
≥65	33/510 (6.5)	18/438 (4.1)	15/72 (20.8)
Sex			
Male	290/510 (56.9)	267/438 (61.0)	23/72 (31.9)
Female	220/510 (43.1)	171/438 (39.0)	49/72 (68.1)
Travel history			
European Union and United Kingdom	48/264 (18.2)	47/223 (21.1)	1/41 (2.4)
United States of America and Canada	29/264 (11.0)	23/223 (10.3)	6/41 (14.6)
Middle East and Iran	3/264 (1.1)	3/223 (1.3)	0/41 (0.0)
China and Japan	1/264 (0.4)	1/223 (0.4)	0/41 (0.0)
Latin America	8/264 (3.0)	8/223 (3.6)	0/41 (0.0)
Other countries	2/264 (0.8)	2/223 (0.9)	0/41 (0.0)
Bahia - Brazilian state	15/264 (5.7)	14/223 (6.3)	1/41 (2.4)
No travel history	158/264 (59.8)	125/223 (56.1)	33/41 (80.5)
Exposure (source of transmission – contact with confirmed or suspected cases)			
Exposure	184/301 (61.1)	157/252 (62.3)	27/49 (55.1)
No exposure	117/301 (38.9)	95/252 (37.7)	22/49 (44.9)
Healthcare professional			
Yes	85/259 (32.8)		
No	174/259 (67.2)		
Smoking history			
Current smoker	6/111 (5.4)	5/89 (5.6)	1/22 (4.5)
Former smoker	15/111 (13.5)	9/89 (10.1)	6/22 (27.3)
Never smoked	90/111 (81.1)	75/89 (84.3)	15/22 (68.2)
Fever on admission			
Yes	61/391 (15.6)	46/322 (14.3)	15/69 (21.7)
No	330/391 (84.4)	276/322 (85.7)	54/69 (78.3)
Median	36.8	36.7	36.8
Temperature distribution on admission			
<37.5°C	296/392 (75.5)	248/322 (77.0)	48/70 (68.6)
37.5-38°C	54/392 (13.8)	44/322 (13.7)	10/70 (14.3)
38.1-39°C	40/392 (10.2)	28/322 (8.7)	12/70 (17.1)
>39°C	2/392 (0.5)	2/322 (0.6)	0/70 (0.0)
Symptoms			
Nasal congestion	216/510 (42.4)	200/438 (45.7)	16/72 (22.2)
Headache	121/510 (23.7)	103/438 (23.5)	18/72 (25.0)
Cough	212/510 (41.6)	195/438 (44.5)	17/72 (23.6)
Sore throat	141/510 (27.6)	127/438 (29.0)	14/72 (19.4)
Sputum production	5/510 (1.0)	3/438 (0.7)	2/72 (2.8)
Fatigue	69/510 (13.5)	56/438 (12.8)	13/72 (18.1)
Dyspnea	40/510 (7.8)	32/438 (7.3)	8/72 (11.1)
Nausea or vomiting	9/510 (1.8)	6/438 (1.4)	3/72 (4.2)
Diarrhea	22/510 (4.3)	20/438 (4.6)	2/72 (2.8)
Myalgia or arthralgia	185/510 (36.3)	157/438 (35.8)	28/72 (38.9)
Chills	22/510 (4.3)	21/438 (4.8)	1/72 (1.4)
Fever	344/510 (67.5)	286/438 (65.3)	58/72 (80.6)
Conjunctival congestion	2/510 (0.4)	2/438 (0.5)	0/72 (0.0)
Other symptoms	12/510 (2.4)	10/438 (2.3)	2/72 (2.8)
No symptoms	18/510 (3.5)	18/438 (4.1)	0/72 (0.0)
Symptoms duration, days			
Mean±SD	2.8±2.2	2.6±2.1	3.9±2.7
Median	2	2	3
Minimum-Maximum	1-15	1-15	1-12
Signs of infection			
Throat congestion	85/484 (17.6)	68/412 (16.5)	17/72 (23.6)
Tonsil swelling	7/484 (1.4)	3/412 (0.7)	4/72 (5.6)
Skin rash	3/484 (0.6)	2/412 (0.5)	1/72 (1.4)
Other alterations	35/484 (7.2)	20/412 (4.9)	15/72 (20.8)
No alterations	390/484 (80.6)	344/412 (83.5)	46/72 (63.9)
Coexisting disorders			
Any coexisting disorder	101/501 (20.2)	65/429 (15.2)	36/72 (50.0)
Asthma or chronic pulmonary obstructive disorder	15/501 (3.0)	11/429 (2.6)	4/72 (5.6)
Diabetes	16/501 (3.2)	6/429 (1.4)	10/72 (13.9)
Hypertension	41/501 (8.2)	26/429 (6.1)	15/72 (20.8)
Coronary heart disease or other heart conditions	14/501 (2.8)	6/429 (1.4)	8/72 (11.1)
Cerebrovascular disease	2/501 (0.4)	0/429 (0.0)	2/72 (2.8)
Hepatitis B, C, HIV or other immunodeficiency	2/501 (0.4)	1/429 (0.2)	1/72 (1.4)
Cancer	9/501 (1.8)	4/429 (0.9)	5/72 (6.9)
Chronic renal disease	3/501 (0.6)	1/429 (0.2)	2/72 (2.8)
Organ transplant	0/501 (0.0)	0/429 (0.0)	0/72 (0.0)
Pregnancy	5/501 (1.0)	5/429 (1.2)	0/72 (0.0)
Other coexisting disorders	37/501 (7.4)	13/429 (3.0)	24/72 (33.3)
No coexisting disorders	400/501 (79.8)	364/429 (84.8)	36/72 (50.0)
Mean BMI±SD	26.2±4.7	25.5±4.7	26.9±4.6
Chronic-use medications			
Any medication	77/510 (15.1)	43/438 (9.8)	34/72 (47.2)
Statin	20/510 (3.9)	6/438 (1.4)	14/72 (19.4)
Multivitamin	3/510 (0.6)	1/438 (0.2)	2/72 (2.8)
Antidepressant	16/510 (3.1)	11/438 (2.5)	5/72 (6.9)
Antihypertensive	30/510 (5.9)	12/438 (2.7)	18/72 (25.0)
Antiplatelet or anticoagulant	8/510 (1.6)	3/438 (0.7)	5/72 (6.9)
Thyroid hormones	14/510 (2.7)	6/438 (1.4)	8/72 (11.1)
continue...			
Antidiabetic	16/510 (3.1)	8/438 (1.8)	8/72 (11.1)
Pain killers	10/510 (2.0)	5/438 (1.1)	5/72 (6.9)
Antibiotics	3/510 (0.6)	2/438 (0.5)	1/72 (1.4)
Corticosteroid	3/510 (0.6)	2/438 (0.5)	1/72 (1.4)
Inhaled medications	4/510 (0.8)	3/438 (0.7)	1/72 (1.4)
Other medications	24/510 (4.7)	11/438 (2.5)	13/72 (18.1)
No use of medications	433/510 (84.9)	395/438 (90.2)	38/72 (52.8)
Chronic-use medications, number of medications (distribution)			
Only 1 type of medication	31/77 (40.3)	18/43 (41.9)	13/34 (38.2)
2 types of medications	19/77 (24.7)	15/43 (34.9)	4/34 (11.8)
3 types of medications	13/77 (16.9)	7/43 (16.3)	6/34 (17.6)
4 or more types of medications – polypharmacy	14/77 (18.2)	3/43 (7.0)	11/34 (32.4)
ESI on arrival			
1	0/345 (0.0)	0/276 (0.0)	0/69 (0.0)
2	20/345 (5.8)	4/276 (1.4)	16/69 (23.2)
3	73/345 (21.2)	59/276 (21.4)	14/69 (20.3)
4	250/345 (72.5)	211/276 (76.4)	39/69 (56.5)
5	2/345 (0.6)	2/276 (0.7)	0/69 (0.0)
Destiny after first evaluation			
Discharge to home	442/496 (89.1)		
Admission on general ward	37/496 (7.5)		
Admission on ICU	17/496 (3.4)		
Return to the emergency room after first evaluation			
Sim	82/370 (22.2)		
Não	288/370 (77.8)		

Results expressed by total n/n (%), if not otherwise indicated.

SD: standard deviation; BMI: body mass index; ESI: Emergency Severity Index; ICU: intensive care unit.

Fever was present in only 15.6% of patients upon admission, but 67.5% had a reported history of fever, followed by nasal congestion (42.4%), cough (41.6%) and myalgia or arthralgia (36.3%). The mean duration of symptoms was 2.8 days, which was the same for patients hospitalized or not. Upon admission, the majority of patients (80.6%) had no significant changes on physical examination. Considering all included patients, 20.2% had at least one comorbidity. This rate, however, was far higher in the hospitalized group (50%) when compared with the non-hospitalized group (15.2%); the most common comorbidities were hypertension and diabetes. The distribution of patients in the Emergency Severity Index (ESI) differs between the two groups analyzed, with the hospitalized group showing a higher rate of ESI 2, indicating that the initial severity was greater in this group since the onset of symptoms.

### Radiologic and laboratory findings

Table 2 demonstrates the radiologic and laboratory findings upon admission. Only 7.3% of patients were initially evaluated with chest radiographs, whereas 15.3% were submitted to computed tomography (CT). Of the radiographs performed, 24.3% had some abnormality, while 93.6% of CT scans showed abnormal results. The most common patterns on chest CT were ground-glass opacity (84.6%) and bilateral patchy shadowing (79.5%).

**Table 2:** Radiologic and laboratory findings

Characteristics	Total number of patients (n=510)	Total number of patients	
		Non-hospitalized patients (n=438)	Hospitalized patients (n=72)
Radiologic findings in chest radiograph			
Chest radiograph performed	37/510 (7.3)	37/438 (8.4)	0/72 (0.0)
Abnormalities on chest radiograph	9/37 (24.3)	9/37 (24.3)	0 (0.0)
Ground-glass opacity	2/37 (5.4)	2/37 (5.4)	0 (0.0)
Local patchy shadowing	1/37 (2.7)	1/37 (2.7)	0 (0.0)
Bilateral patchy shadowing	0/37 (0.0)	0/37 (0.0)	0 (0.0)
Interstitial abnormalities	6/37 (16.2)	6/37 (16.2)	0 (0.0)
Radiologic findings in chest CT			
Chest CT performed	78/510 (15.3)	23/438 (5.3)	55/72 (76.4)
Abnormalities on chest CT	73/78 (93.6)	20/23 (87.0)	53/55 (96.4)
Ground glass opacity	66/78 (84.6)	17/23 (73.9)	49/55 (89.1)
Local patchy shadowing	2/78 (2.6)	1/23 (4.3)	1/55 (1.8)
Bilateral patchy shadowing	62/78 (79.5)	17/23 (73.9)	45/55 (81.8)
Interstitial abnormalities	37/78 (47.4)	11/23 (47.8)	26/55 (47.3)
Laboratory findings			
Median PaO _2_ /FiO _2_ ratio (IQR)			3.0 (1.3- 5.5)
White blood cell count			
Median per mm ^3^	5.568	5.321	5.868
Distribution per mm ^3^			
>10.000	6/135 (4.4)	2/63 (3.2)	4/72 (5.6)
<4.000	29/135 (21.5)	14/63 (22.2)	15/72 (20.8)
Lymphocyte count			
Median per mm ^3^	1.213	1.242	1.178
Distribution <1.500 per mm ^3^	103/135 (76.3)	53/63 (84.1)	50/72 (69.4)
Platelet count			
Median per mm ^3^	189.155	189.918	188.229
Distribution <150.000 per mm ^3^	35/135 (25.9)	16/63 (25.4)	19/72 (26.4)
Median hemoglobin, g/dL	14.29	14.47	14.07
Distribution of other findings			
C-reactive protein >5mg/L	91/122 (74.6)	35/62 (56.5)	56/60 (93.3)
Procalcitonin >0.5ng/mL	3/15 (20.0)	0/3 (0.0)	3/12 (25.0)
Lactate dehydrogenase >214U/L	40/70 (57.1)	9/24 (37.5)	31/46 (67.4)
Aspartate aminotransferase >40U/L	15/75 (20.0)	5/27 (18.5)	10/48 (20.8)
Alanine aminotransferase >40U/L	19/75 (25.3)	7/27 (25.9)	12/48 (25.0)
Total bilirubin >1.2mg/dL	3/70 (4.3)	1/24 (4.2)	2/46 (4.3)
Creatine kinase >180 U/L	5/53 (9.4)	2/16 (12.5)	3/37 (8.1)
Creatinine >1mg/dL	28/66 (42.4)	15/35 (42.9)	13/31 (41.9)
D-dimer >500ng/mL	30/78 (38.5)	7/24 (29.2)	23/54 (42.6)
Mean sodium, mmol/L	138.52	139.13	138.21
Mean potassium, mmol/L	4.16	4.17	4.15

Results expressed by total n/n (%), if not otherwise indicated.

CT: computer tomography; PaO _2_ /FiO _2_ : oxygen partial pressure/fractional inspired oxygen; IQR: interquartile range.

Upon admission, lymphocytopenia was identified in 76.3% of patients, thrombocytopenia in 25.9%, and leukopenia in 21.5%. Most patients had elevated levels of both C-reactive protein and lactate dehydrogenase. Less common findings were elevated levels of D-dimer, aspartate aminotransferase and alanine aminotransferase. The hospitalized group had more patients with higher levels of C-reactive protein, procalcitonin and lactate dehydrogenase. The other results do not show any major difference between groups. A viral panel was collected in 146 (29%) patients, and it was positive for rhinovirus in nine cases, influenza B in two cases, and influenza A, in one case.

### Treatment and complications

As shown in table 3 , 72 (14%) patients had been hospitalized at HIAE by the time of the analysis. Among those, 20 patients (27.8%) required intensive care during their hospital stay; in that, 12 were referred from the emergency room to the ICU, and eight presented worsening of the clinical condition at inpatients units and were transferred to the ICU. The majority of patients received intravenous antibiotic therapy (84.7%), 45.8% received chloroquine and 31.9% oseltamivir. Oxygen therapy was necessary in 44.4% of hospitalized patients; 23.6% required mechanical ventilation (18.1% invasive and 5.6% non-invasive) and extracorporeal membrane oxygenation (ECMO) was used in only one case. Considering patients admitted to the ICU, invasive mechanical ventilation was required by 65% of them. During hospital admission, most patients were diagnosed with pneumonia (58.3%), followed by acute kidney injury (9.7%) and ARDS (8.3%). The mean LOS was 9 days; considering only patients requiring intensive care, the mean ICU LOS was 15.25 days, and the mean total LOS was 22 days, whereas for patients not admitted to the ICU, the mean LOS was 7 days. Only one patient died in this series, that is, 1.38% mortality rate.

**Table 3:** Treatments, complications and clinical outcomes

Characteristic	Total number of patients (n=510)
Disease severity	
Severe	18/441 (4.1)
Not severe	423/441 (95.9)
Intensive care use during hospital stay	
Yes	20/72 (27.8)
No	52/72 (72.2)
Hospital treatments – medications	
Intravenous antibiotics	61/72 (84.7)
Oseltamivir	23/72 (31.9)
Lopinavir and ritonavir	16/72 (22.2)
Chloroquine	33/72 (45.8)
Corticosteroids	1/72 (1.4)
Hospital treatments – support treatments	
Oxygen therapy	32/72 (44.4)
Mechanical ventilation	17/72 (23.6)
Invasive	13/72 (18.1)
Non-invasive	4/72 (5.6)
Extracorporeal membrane oxygenation	1/72 (1.4)
Continuous renal replacement therapy	3/72 (4.2)
Complications	
Septic shock	5/72 (6.9)
Acute respiratory distress syndrome	6/72 (8.3)
Acute kidney injury	7/72 (9.7)
Pneumonia	42/72 (58.3)
Mean length of stay, days	
LOS, all patients	9
Patients requiring ICU, days	
ICU	15.25
Inpatients units	6.75
Patients not requiring ICU, inpatients units, days	7

Results expressed by total n/n (%), if not otherwise indicated.

LOS: length of stay; ICU: intensive care unit.

## DISCUSSION

It took 3 months from the first diagnosed case of COVID-19 in China until diagnosis of patient zero in Brazil, on February 26, 2020, at HIAE. During 16 days after the first diagnosis, all cases had a history of recent international travels. On March 11, 2020, the first case of local transmission was confirmed, also at HIAE. A relevant proportion of all patients with confirmed COVID-19 infection had been diagnosed at HIAE by the time of the analysis.

The patients in our series had a mean age of 39.9 years and were mostly male (56.9%). The studies describing demographic characteristics in the infected general population showed a median age of 47 years, ^( 7 , 12 )^ and the proportion of males was 58.1% in the Chinese report ^( 7 )^ and 50% in the Singapore report. ^( 12 )^ The respiratory symptoms were similar to those of patients described in reports from China, United States and Europe. ^( 7 , 9 , 13 )^ However, the mean days of symptoms was far lower in our series (2.8 days *versus* 13 days in Singapore, ^( 12 )^ 7 days in the United States ^( 13 )^ and 7 days in China. ^( 3 )^

Although fever was reported by the majority of patients, it was only present in 15.6% of patients at the initial assessment at hospital, suggesting not only it might not be considered to determine severity of illness, but also that diagnostic algorithms using fever for testing may mask the total number of cases and delay diagnosis. The prevalence of chronic diseases was far higher in the hospitalized group (50%) as compared to non-hospitalized group (15.2%). This prevalence was even higher in the subgroup admitted to the ICU (80%).

The mean age of hospitalized patients was higher than non-hospitalized patients (51.8 *versus* 38.6 years) and the required hospitalization increased with age (7.8% for patients aged 12 to 49 years, 33.8% for 50 to 64 years, and 45.5% for patients older than 65 years). In this Brazilian case series, hospitalization was required for 72 (14.1%) patients, and 20 of them demanded critical care, accounting for 27.8% of total admissions, a number far greater than the Chinese series, in which only 5% required ICU. ^( 7 )^

The majority of patients were admitted to the ICU because of acute hypoxemic respiratory failure that required ventilatory support. Invasive mechanical ventilation was needed in 65% of ICU patients (18.1% of total hospitalizations), whereas 20% were managed with non-invasive mechanical ventilation. The necessity of invasive mechanical ventilation was similar to an ICU series reported from the United States (75% of Washington), ^( 13 )^ lower than that reported in an Italian publication (88% of Lombardy), ^( 9 )^ but higher than the Chinese reports (47%, 42% and 30% of Wuhan; half of these treated with extracorporeal membrane oxygenation). ^( 3 , 14 , 15 )^ Considering the use of non-invasive ventilation, the rate was again similar to that reported in Washington (19%) ^( 12 )^ and lower than the rates in China (42%, 56% and 62% of Wuhan, including patients receiving high-flow nasal cannula). ^( 3 , 14 , 15 )^ A total of three patients (15% of patients admitted to the ICU) developed acute kidney injury and required continuous renal replacement therapy. Among those, only one patient had chronic kidney disease. The prevalence of chronic kidney disease was 2.9% among hospitalized patients in the Chinese report, ^( 14 )^ and 21% among patients admitted to the ICU in the series from the United States (21%). ^( 13 )^

This study has important limitations. First, part of the cases had incomplete information documented in the medical records, and patient clinical history documentation was not homogeneous among all patients. This is a common limitation in retrospective observational studies, taking into account that data generation was clinically driven and not in systematic fashion. Second, since many patients remained at the hospital and the outcomes were unknown at the time of data collection, we censored the data regarding their clinical outcomes as of the time of the analysis. Third, only patients hospitalized at HIAE were included in the hospitalization group, and there is no documentation of hospital admissions outside of our service network. Finally, this study only included patients attended as outpatients or inpatients at HIAE; therefore, asymptomatic and mild cases who did not seek medical care were not considered. Hence, our study cohort may represent more severe COVID-19 cases.

## CONCLUSION

To date, there is no study in Brazil reporting the characteristics of patients diagnosed with COVID-19. Brazil is the country in the south hemisphere with the highest number of confirmed cases this disease and *Hospital Israelita Albert Einstein* is the center where the first patient was diagnosed, with a representative sample of all confirmed COVID-19 cases in the country. The results presented in this study may be relevant for Brazil and other countries with similar characteristics, which are starting to deal with this pandemic.
