# Supplementary material for: Epidemiologic and clinical features of patients with COVID-19 in Brazil
Source: Einstein (Sao Paulo). 2020 Aug 12;18:eAO6022. doi: 10.31744/einstein_journal/2020AO6022 (PMC7422909; doi:10.31744/einstein_journal/2020AO6022)
Supplement: Translation

# Translation: Características epidemiológicas e clínicas dos pacientes com COVID-19 no Brasil

**Authors:** Vanessa Damazio Teich, Sidney Klajner, Felipe Augusto Santiago de Almeida, Anna Carolina Batista Dantas, Claudia Regina Laselva, Mariana Galvani Torritesi, Tatiane Ramos Canero, Otávio Berwanger, Luiz Vicente Rizzo, Eduardo Pontes Reis, Miguel Cendoroglo

**Affiliations:** 1 São PauloSP Brazil Hospital Israelita Albert Einstein, São Paulo , SP , Brazil .

**Keywords:** Doenças transmissíveis, Pneumopatias/epidemiologia, SARS-CoV-2, COVID-19, Infecções por coronavírus, Epidemiologia

## Abstract

**Objetivo:**

Descrever as características epidemiológicas e clínicas de pacientes com infecção confirmada pelo SARS-CoV-2, diagnosticados e tratados no Hospital Israelita Albert Einstein, que admitiu os primeiros pacientes com essa condição no Brasil.

**Métodos:**

Neste estudo retrospectivo, de centro único, incluímos todos os casos com confirmação laboratorial de COVID-19 no Hospital Israelita Albert Einstein, em São Paulo (SP) de fevereiro a março de 2020. Foram analisados dados demográficos, clínicos, laboratoriais e radiológicos.

**Resultados:**

Foram incluídos 510 pacientes com diagnóstico confirmado de COVID-19. A maioria dos pacientes era do sexo masculino (56,9%), com média de idade de 40 anos. Foi relatada história de contato próximo com um caso positivo/suspeito por 61,1% dos pacientes, e 34,4% tinham história de viagens internacionais recentes. Os sintomas mais comuns foram febre (67,5%), congestão nasal (42,4%), tosse (41,6%) e mialgia/artralgia (36,3%). A tomografia computadorizada de tórax foi realizada em 78 (15,3%) pacientes, e 93,6% deles apresentaram resultados anormais. A hospitalização foi necessária para 72 (14%) pacientes, e 20 (27,8%) foram admitidos na Unidade de Terapia Intensiva. Quanto ao tratamento clínico, os medicamentos mais utilizados foram antibióticos intravenosos (84,7%), cloroquina (45,8%) e oseltamivir (31,9%). A ventilação mecânica invasiva foi necessária em 65% dos pacientes na Unidade de Terapia Intensiva. O tempo médio de internação foi 9 dias para todos os pacientes (22 e 7 dias para pacientes que necessitaram ou não de cuidados intensivos, respectivamente). Apenas um (1,38%) paciente morreu durante o acompanhamento.

**Conclusão:**

Estes resultados podem ser relevantes para o Brasil e outros países com características semelhantes, que começaram a lidar com essa pandemia.

## INTRODUÇÃO

Desde dezembro de 2019, vários casos de pneumonia de origem desconhecida foram relatados em Wuhan, na China.^( 1 )^O patógeno foi então identificado como um novo coronavírus, um vírus de RNA, atualmente denominado coronavírus da síndrome respiratória aguda grave 2 (SARS-CoV-2).^( 2 )^Huang et al., relataram os primeiros casos na China, com manifestação clínica comum de febre, tosse, mialgia, fadiga e dispneia, com disfunção de órgãos (por exemplo: síndrome do desconforto respiratório agudo – SDRA –, choque, cardiopatias e lesões renais agudas) e morte, nos casos graves.^( 3 )^

Posteriormente, em janeiro de 2020, a Organização Mundial da Saúde (OMS) declarou o surto uma Emergência de Saúde Pública de Interesse Internacional (ESPII) e, depois, em março de 2020, foi caracterizado como uma pandemia.^( 4 )^Em 7 de abril de 2020, 1.429.437 casos tinham sido relatados em 184 países e regiões nos cinco continentes, com 82.074 mortes em todo o mundo.^( 5 )^Mais recentemente, o Centro de Controle e Prevenção de Doenças Chinês publicou dados de 72.314 pacientes, sendo 44.672 (62%) classificados como casos confirmados da doença causada pelo coronavírus 2019 (COVID-19). A maioria dos pacientes tinha 30 a 79 anos (87%), com apresentação clínica leve (81%; ou seja, sem pneumonia e com pneumonia leve) e letalidade geral de 2,3% (maior na população idosa, com taxa de letalidade de 14,8% nos pacientes com 80 anos ou mais).^( 6 )^

Em 26 de fevereiro de 2020, o primeiro paciente brasileiro teve um diagnóstico confirmado de COVID-19 no Hospital Israelita Albert Einstein (HIAE). O HIAE é um hospital filantrópico localizado na cidade de São Paulo (SP), com doze unidades de saúde, incluindo um hospital quaternário com 592 leitos e quatro unidades ambulatoriais de emergência. Ao final deste estudo, em 25 de março de 2020, dentre os 2.433 pacientes com COVID-19 confirmada no Brasil, 32% (769) tinham sido diagnosticados no HIAE.

Dada a rápida disseminação da COVID-19, os dados clínicos e epidemiológicos de vários países são publicados diariamente.^( 7 - 9 )^No entanto, nenhum estudo foi relatado até o momento apresentando as características dos pacientes com COVID-19 diagnosticados no Brasil.

## OBJETIVO

Descrever as características epidemiológicas e clínicas de pacientes com infecção confirmada pelo SARS-CoV-2, diagnosticados e tratados no Hospital Israelita Albert Einstein, que admitiu os primeiros pacientes com essa doença no Brasil.

## MÉTODOS

### Desenho e supervisão do estudo

Trata-se de estudo observacional retrospectivo, de centro único, que incluiu todos os pacientes consecutivos com diagnóstico confirmado de COVID-19, no HIAE, entre 26 de fevereiro de 2020 e 25 de março de 2020. O estudo foi financiado por recursos próprios do HIAE e elaborado pelos pesquisadores. O estudo foi aprovado pelo Comitê de Ética em Pesquisa da instituição, número do parecer 3.921.190, CAAE: 30047620.3.0000.0071, e pela Comissão Nacional de Ética em Pesquisa (Conep).

### Pacientes

O diagnóstico da COVID-19 foi realizado de acordo com as orientações provisórias da OMS.^( 10 )^Um caso confirmado de COVID-19 foi definido como um resultado positivo da reação em cadeia de polimerase via transcriptase reversa em tempo real (RT-PCR) de amostras de *swab* nasais e faríngeos.^( 11 )^Todos os casos incluídos na análise atual tiveram confirmação laboratorial.

### Fontes de dados

Os dados foram obtidos no prontuário eletrônico do paciente (PEP), incluindo pacientes internados e ambulatoriais com COVID-19 confirmada por testes laboratoriais. Os dados coletados incluíram informações demográficas, clínicas, laboratoriais e radiológicas e foram anonimizados, para que os pacientes não pudessem ser identificados.

As características demográficas incluíram idade, sexo, tabagismo, peso e índice de massa corporal (IMC). As informações clínicas incluíam história pregressa, histórico de viagens e exposição, sinais, sintomas, doenças de base, uso contínuo de medicamentos e medidas terapêuticas (ou seja, terapia antiviral, corticoterapia, suporte respiratório e terapia de reposição renal). Hemograma completo, avaliação das funções renal e hepática e medidas de eletrólitos, dímero D, procalcitonina, lactato desidrogenase, proteína C-reativa e creatina quinase fizeram parte da avaliação laboratorial. As alterações radiológicas foram definidas com base no relatório médico documentado no PEP. A duração da doença desde o início dos sintomas e o tempo de permanência no hospital (TPH) e na Unidade de Terapia Intensiva (UTI) também foram documentados.

### Análise estatística

As variáveis contínuas foram expressas em média com desvio padrão, mediana, valores mínimo e máximo. As variáveis categóricas foram resumidas em contagens e percentagens. Nenhuma imputação foi feita para dados ausentes. Todas as estatísticas são consideradas apenas descritivas, pois a coorte de pacientes em nosso estudo não foi derivada de seleção aleatória. Todas as análises foram realizadas com o uso do Microsoft Excel 2013.

## RESULTADOS

### Características demográficas e clínicas

Entre 26 de fevereiro e 25 de março de 2020, 769 pacientes foram diagnosticados com COVID-19 no HIAE. Este estudo incluiu 510 (66%) pacientes, cujos dados relacionados a demografia, sintomas clínicos, exames laboratoriais e de imagens estavam disponíveis no PEP da instituição. Os 259 pacientes restantes usaram apenas as instalações laboratoriais do hospital, tendo acompanhamento médico com profissionais de fora da rede.

As características demográficas e clínicas dos pacientes são mostradas na tabela 1 . Dentre os pacientes, 34,4% tinham história recente de viagens internacionais, e 5,7% tinham estado em uma mesma festa de casamento na Bahia, um estado da região nordeste do Brasil; 184 (61,1%) pacientes tinham história de contato próximo com um caso positivo ou suspeito de COVID-19. A maioria dos pacientes era do sexo masculino (56,9%), e a média de idade foi 40 anos. Apenas 0,6% dos pacientes tinham menos de 11 anos, e 6,5% tinham mais de 65 anos.

**Tabela 1:** Caraterísticas clínicas e epidemiológicas

Característica	Total de pacientes (n=510)	Total de pacientes	
		Não hospitalizados (n=438)	Hospitalizados (n=72)
Idade, anos			
Média±DP	39,9±13,6	38,6±12,5	51,8±15,32
Mediana	38,0	37,0	52,0
Mínima-Máxima	2-92	2-84	5-92
Número de pacientes	510	438	72
Distribuição segundo idade, anos			
0-11	3/510 (0,6)	2/438 (0,5)	1/72 (1,4)
12-49	400/510 (78,4)	369/438 (84,2)	31/72 (43,1)
50-64	74/510 (14,5)	49/438 (11,2)	25/72 (34,7)
≥65	33/510 (6,5)	18/438 (4,1)	15/72 (20,8)
Sexo			
Masculino	290/510 (56,9)	267/438 (61,0)	23/72 (31,9)
Feminino	220/510 (43,1)	171/438 (39,0)	49/72 (68,1)
História de viagem			
União Europeia e Reino Unido	48/264 (18,2)	47/223 (21,1)	1/41 (2,4)
Estados Unidos e Canadá	29/264 (11,0)	23/223 (10,3)	6/41 (14,6)
Oriente Médio e Irã	3/264 (1,1)	3/223 (1,3)	0/41 (0,0)
China e Japão	1/264 (0,4)	1/223 (0,4)	0/41 (0,0)
América Latina	8/264 (3,0)	8/223 (3,6)	0/41 (0,0)
Outros países	2/264 (0,8)	2/223 (0,9)	0/41 (0,0)
Bahia – estado brasileiro	15/264 (5,7)	14/223 (6,3)	1/41 (2,4)
Sem história de viagem	158/264 (59,8)	125/223 (56,1)	33/41 (80,5)
Exposição (fonte de transmissão – contato com casos confirmados ou suspeitos)			
Exposição	184/301 (61,1)	157/252 (62,3)	27/49 (55,1)
Sem exposição	117/301 (38,9)	95/252 (37,7)	22/49 (44,9)
Profissional de saúde			
Sim	85/259 (32,8)		
Não	174/259 (67,2)		
História de tabagismo			
Fumante atual	6/111 (5,4)	5/89 (5,6)	1/22 (4,5)
Ex-fumante	15/111 (13,5)	9/89 (10,1)	6/22 (27,3)
Nunca fumou	90/111 (81,1)	75/89 (84,3)	15/22 (68,2)
Febre na admissão			
Sim	61/391 (15,6)	46/322 (14,3)	15/69 (21,7)
Não	330/391 (84,4)	276/322 (85,7)	54/69 (78,3)
Mediana	36,8	36,7	36,8
Distribuição de temperatura na admissão			
<37,5^°^C	296/392 (75,5)	248/322 (77,0)	48/70 (68,6)
37,5-38^°^C	54/392 (13,8)	44/322 (13,7)	10/70 (14,3)
38,1-39^°^C	40/392 (10,2)	28/322 (8,7)	12/70 (17,1)
>39^°^C	2/392 (0,5)	2/322 (0,6)	0/70 (0,0)
Sintomas			
Congestão nasal	216/510 (42,4)	200/438 (45,7)	16/72 (22,2)
Cefaleia	121/510 (23,7)	103/438 (23,5)	18/72 (25,0)
Tosse	212/510 (41,6)	195/438 (44,5)	17/72 (23,6)
Dor de garganta	141/510 (27,6)	127/438 (29,0)	14/72 (19,4)
Produção de escarro	5/510 (1,0)	3/438 (0,7)	2/72 (2,8)
Fadiga	69/510 (13,5)	56/438 (12,8)	13/72 (18,1)
Dispneia	40/510 (7,8)	32/438 (7,3)	8/72 (11,1)
Náusea ou vômitos	9/510 (1,8)	6/438 (1,4)	3/72 (4,2)
Diarreia	22/510 (4,3)	20/438 (4,6)	2/72 (2,8)
Mialgia ou artralgia	185/510 (36,3)	157/438 (35,8)	28/72 (38,9)
Calafrios	22/510 (4,3)	21/438 (4,8)	1/72 (1,4)
Febre	344/510 (67,5)	286/438 (65,3)	58/72 (80,6)
Congestão conjuntival	2/510 (0,4)	2/438 (0,5)	0/72 (0,0)
Outros sintomas	12/510 (2,4)	10/438 (2,3)	2/72 (2,8)
Sem sintomas	18/510 (3,5)	18/438 (4,1)	0/72 (0,0)
Duração dos sintomas, dias			
Média±DP	2,8±2,2	2,6±2,1	3,9±2,7
Mediana	2	2	3
Mínima-Máxima	1-15	1-15	1-12
Sinais de infecção			
Garganta inflamada	85/484 (17,6)	68/412 (16,5)	17/72 (23,6)
Amígdalas inchadas	7/484 (1,4)	3/412 (0,7)	4/72 (5,6)
Erupções cutâneas	3/484 (0,6)	2/412 (0,5)	1/72 (1,4)
Outras alterações	35/484 (7,2)	20/412 (4,9)	15/72 (20,8)
Sem alterações	390/484 (80,6)	344/412 (83,5)	46/72 (63,9)
Doenças coexistentes			
Quaisquer doenças coexistentes	101/501 (20,2)	65/429 (15,2)	36/72 (50,0)
Asma ou doença pulmonar obstrutiva crônica	15/501 (3,0)	11/429 (2,6)	4/72 (5,6)
Diabetes	16/501 (3,2)	6/429 (1,4)	10/72 (13,9)
Hipertensão	41/501 (8,2)	26/429 (6,1)	15/72 (20,8)
Doença coronariana ou outras doenças cardíacas	14/501 (2,8)	6/429 (1,4)	8/72 (11,1)
Doenças cerebrovasculares	2/501 (0,4)	0/429 (0,0)	2/72 (2,8)
Hepatite B, C, HIV ou outra imunodeficiência	2/501 (0,4)	1/429 (0,2)	1/72 (1,4)
Câncer	9/501 (1,8)	4/429 (0,9)	5/72 (6,9)
Doença renal crônica	3/501 (0,6)	1/429 (0,2)	2/72 (2,8)
Transplante de órgão	0/501 (0,0)	0/429 (0,0)	0/72 (0,0)
Gravidez	5/501 (1,0)	5/429 (1,2)	0/72 (0,0)
Outras doenças coexistentes	37/501 (7,4)	13/429 (3,0)	24/72 (33,3)
Sem doenças coexistentes	400/501 (79,8)	364/429 (84,8)	36/72 (50,0)
IMC médio±DP	26,2±4,7	25,5±4,7	26,9±4,6
Uso crônico de medicamentos			
Quaisquer medicamentos	77/510 (15,1)	43/438 (9,8)	34/72 (47,2)
Estatinas	20/510 (3,9)	6/438 (1,4)	14/72 (19,4)
Polivitaminas	3/510 (0,6)	1/438 (0,2)	2/72 (2,8)
Antidepressivos	16/510 (3,1)	11/438 (2,5)	5/72 (6,9)
Anti-hipertensivos	30/510 (5,9)	12/438 (2,7)	18/72 (25,0)
Antiplaquetários ou anticoagulantes	8/510 (1,6)	3/438 (0,7)	5/72 (6,9)
Hormônios tireoidianos	14/510 (2,7)	6/438 (1,4)	8/72 (11,1)
Antidiabéticos	16/510 (3,1)	8/438 (1,8)	8/72 (11,1)
Analgésicos	10/510 (2,0)	5/438 (1,1)	5/72 (6,9)
Antibióticos	3/510 (0,6)	2/438 (0,5)	1/72 (1,4)
Corticoides	3/510 (0,6)	2/438 (0,5)	1/72 (1,4)
Medicamentos inalados	4/510 (0,8)	3/438 (0,7)	1/72 (1,4)
Outros medicamentos	24/510 (4,7)	11/438 (2,5)	13/72 (18,1)
Sem uso de medicamentos	433/510 (84,9)	395/438 (90,2)	38/72 (52,8)
Uso crônico de medicamentos, número de medicamentos (distribuição)			
Somente um tipo de medicamento	31/77 (40,3)	18/43 (41,9)	13/34 (38,2)
Dois tipos de medicamentos	19/77 (24,7)	15/43 (34,9)	4/34 (11,8)
Três tipos de medicamentos	13/77 (16,9)	7/43 (16,3)	6/34 (17,6)
Quatro ou mais tipos de medicamentos – polifarmácia	14/77 (18,2)	3/43 (7,0)	11/34 (32,4)
ESI na chegada			
1	0/345 (0,0)	0/276 (0,0)	0/69 (0,0)
2	20/345 (5,8)	4/276 (1,4)	16/69 (23,2)
3	73/345 (21,2)	59/276 (21,4)	14/69 (20,3)
4	250/345 (72,5)	211/276 (76,4)	39/69 (56,5)
5	2/345 (0,6)	2/276 (0,7)	0/69 (0,0)
Destino após primeira avaliação			
Alta para casa	442/496 (89,1)		
Admissão em enfermaria geral	37/496 (7,5)		
Admissão em UTI	17/496 (3,4)		
Retorno ao pronto-socorro após primeira avaliação			
Sim	82/370 (22,2)		
Não	288/370 (77,8)		

Resultados expressos por n/n total (%), se não indicado de outra forma.

DP: desvio padrão; IMC: índice de massa corpórea; ESI: Índice de Gravidade de Emergência; UTI: unidade de terapia intensiva.

A febre estava presente em apenas 15,6% dos pacientes na admissão, mas 67,5% tinham história relatada de febre, seguida de congestão nasal (42,4%), tosse (41,6%) e mialgia ou artralgia (36,3%). A duração média dos sintomas foi de 2,8 dias – a mesma entre os pacientes internados ou não. Na admissão, a maioria dos pacientes (80,6%) não apresentou alterações significativas ao exame físico. Considerando todos os pacientes incluídos, 20,2% apresentavam pelo menos uma comorbidade. Essa taxa, no entanto, foi muito maior no grupo hospitalizado (50%) quando comparado ao não hospitalizado (15,2%); as comorbidades mais comuns foram hipertensão e diabetes. A distribuição dos pacientes pelo Índice de Gravidade de Emergência (ESI) difere entre os dois grupos analisados, com o grupo hospitalizado apresentando um índice mais alto (ESI 2), o que indica maior gravidade inicial nesse grupo desde o aparecimento dos sintomas.

### Achados radiológicos e laboratoriais

A tabela 2 mostra os achados radiológicos e laboratoriais na admissão. Apenas 7,3% dos pacientes foram avaliados inicialmente com radiografia de tórax, ao passo que 15,3% foram submetidos à tomografia computadorizada (TC). Das radiografias realizadas, 24,3% apresentaram alguma alteração, enquanto 93,6% das TC mostraram resultados anormais. Os padrões mais comuns na TC de tórax foram opacidade em vidro fosco (84,6%) e consolidações bilaterais irregulares (79,5%).

**Tabela 2:** Achados radiológicos e laboratoriais

Características	Total de pacientes (n=510)	Total de pacientes	
		Pacientes não hospitalizados (n=438)	Pacientes hospitalizados (n=72)
Achados radiológicos em radiografia de tórax			
Radiografia de tórax realizada	37/510 (7,3)	37/438 (8,4)	0/72 (0,0)
Alterações na radiografia de tórax	9/37 (24,3)	9/37 (24,3)	0 (0,0)
Opacidade em vidro fosco	2/37 (5,4)	2/37 (5,4)	0 (0,0)
Consolidações focais	1/37 (2,7)	1/37 (2,7)	0 (0,0)
Consolidações bilaterais	0/37 (0,0)	0/37 (0,0)	0 (0,0)
Alterações intersticiais	6/37 (16,2)	6/37 (16,2)	0 (0,0)
Achados radiológicos em TC de tórax			
TC de tórax realizada	78/510 (15,3)	23/438 (5,3)	55/72 (76,4)
Alterações na TC de tórax	73/78 (93,6)	20/23 (87,0)	53/55 (96,4)
Opacidade em vidro fosco	66/78 (84,6)	17/23 (73,9)	49/55 (89,1)
Consolidações focais	2/78 (2,6)	1/23 (4,3)	1/55 (1,8)
Consolidações bilaterais	62/78 (79,5)	17/23 (73,9)	45/55 (81,8)
Alterações intersticiais	37/78 (47,4)	11/23 (47,8)	26/55 (47,3)
Achados laboratoriais			
PaO_2_/FiO_2_mediana (IIQ)			3,0 (1,3- 5,5)
Contagem de leucócitos			
Mediana por mm^3^	5,568	5,321	5,868
Distribuição por mm^3^			
>10.000	6/135 (4,4)	2/63 (3,2)	4/72 (5,6)
<4.000	29/135 (21,5)	14/63 (22,2)	15/72 (20,8)
Contagem de linfócitos			
Mediana por mm^3^	1,213	1,242	1,178
Distribuição <1.500 por mm^3^	103/135 (76,3)	53/63 (84,1)	50/72 (69,4)
Contagem de plaquetas			
Mediana por mm^3^	189,155	189,918	188,229
Distribuição <150.000 por mm^3^	35/135 (25,9)	16/63 (25,4)	19/72 (26,4)
Hemoglobina mediana, g/dL	14,29	14,47	14,07
Distribuição de outros achados			
Proteína C-reativa >5mg/L	91/122 (74,6)	35/62 (56,5)	56/60 (93,3)
Procalcitonina >0,5ng/mL	3/15 (20,0)	0/3 (0,0)	3/12 (25,0)
Lactato desidrogenase >214U/L	40/70 (57,1)	9/24 (37,5)	31/46 (67,4)
Aspartato aminotransferase >40U/L	15/75 (20,0)	5/27 (18,5)	10/48 (20,8)
Alanina aminotransferase >40U/L	19/75 (25,3)	7/27 (25,9)	12/48 (25,0)
Bilirrubinas totais >1,2mg/dL	3/70 (4,3)	1/24 (4,2)	2/46 (4,3)
Creatina quinase >180U/L	5/53 (9,4)	2/16 (12,5)	3/37 (8,1)
Creatinina >1mg/dL	28/66 (42,4)	15/35 (42,9)	13/31 (41,9)
Dímero D >500ng/mL	30/78 (38,5)	7/24 (29,2)	23/54 (42,6)
Sódio médio, mmol/L	138,52	139,13	138,21
Potássio médio, mmol/L	4,16	4,17	4,15

Resultados expressos por n/n total (%), se não indicado de outra forma.

TC: tomografia computadorizada; PaO_2_/FiO_2_: pressão parcial de oxigênio/fração inspirada de oxigênio; IIQ: intervalo interquartil.

Na admissão, foi identificada linfocitopenia em 76,3% dos pacientes, trombocitopenia em 25,9% e leucopenia em 21,5%. A maioria dos pacientes apresentou níveis elevados de proteína C-reativa e lactato desidrogenase. Os achados menos comuns foram os níveis elevados de dímero D, aspartato aminotransferase e alanina aminotransferase. O grupo hospitalizado teve mais pacientes com níveis mais altos de proteína C-reativa, procalcitonina e lactato desidrogenase. Os outros resultados não mostraram grande diferença entre os grupos. Um painel viral foi coletado em 146 (29%) pacientes, sendo positivo para rinovírus em nove casos, influenza B em dois casos e influenza A em um caso.

### Tratamento e complicações

Conforme mostrado na tabela 3 , 72 (14%) pacientes tinham sido hospitalizados no HIAE no momento da análise. Entre estes, 20 (27,8%) necessitaram de cuidados intensivos durante a internação; 12 foram diretamente da sala de emergência para a UTI, ao passo que os outros oito tiveram piora do quadro em unidades de internação e foram transferidos para a UTI. A maioria dos pacientes recebeu antibioticoterapia intravenosa (84,7%), 45,8% receberam cloroquina e 31,9% oseltamivir. A oxigenoterapia foi necessária em 44,4% dos pacientes hospitalizados; 23,6% demandaram ventilação mecânica (18,1% invasiva e 5,6% não invasiva) e em apenas um caso foi utilizada oxigenação por membrana extracorpórea (ECMO). Considerando os pacientes admitidos na UTI, a ventilação mecânica invasiva foi necessária em 65% deles. Durante a internação hospitalar, a maioria dos pacientes foi diagnosticada com pneumonia (58,3%), seguida de lesão renal aguda (9,7%) e SDRA (8,3%). O período médio de internação foi 9 dias; considerando apenas os pacientes que necessitaram de cuidados intensivos, o tempo médio de permanência na UTI foi 15,25 dias, e o tempo médio total de internação foi 22 dias, ao passo que, para os pacientes que não necessitaram de UTI, o tempo médio de internação foi 7 dias. Apenas um paciente morreu nesta série, representando taxa de mortalidade de 1,38%.

**Tabela 3:** Tratamentos, complicações e desfechos clínicos

Característica	Total de pacientes (n=510)
Gravidade da doença	
Grave	18/441 (4,1)
Não grave	423/441 (95,9)
Uso de terapia intensiva durante hospitalização	
Sim	20/72 (27,8)
Não	52/72 (72,2)
Tratamentos hospitalares – medicamentos	
Antibióticos intravenosos	61/72 (84,7)
Oseltamivir	23/72 (31,9)
Lopinavir e ritonavir	16/72 (22,2)
Cloroquina	33/72 (45,8)
Corticoides	1/72 (1,4)
Tratamentos hospitalares – tratamento de suporte	
Oxigenoterapia	32/72 (44,4)
Ventilação mecânica	17/72 (23,6)
Invasiva	13/72 (18,1)
Não invasiva	4/72 (5,6)
Oxigenação por membrana extracorpórea	1/72 (1,4)
Terapia de substituição renal contínua	3/72 (4,2)
Complicações	
Choque séptico	5/72 (6,9)
Síndrome de desconforto respiratório agudo	6/72 (8,3)
Lesão renal aguda	7/72 (9,7)
Pneumonia	42/72 (58,3)
Tempo médio de internação, dias	
Tempo de internação, todos os pacientes	9
Pacientes que precisaram de UTI, dias	
UTI	15,25
Unidade de internação	6,75
Pacientes que não precisaram de UTI, unidade de internação, dias	7

Resultados expressos por n/n total (%), se não indicado de outra forma.

UTI: unidade de terapia intensiva.

## DISCUSSÃO

Quase 3 meses se passaram desde o primeiro caso diagnosticado de COVID-19 na China até o paciente zero ser diagnosticado no Brasil, em 26 de fevereiro de 2020, no HIAE. Nos 16 dias subsequentes ao primeiro diagnóstico, todos os casos tinham história de viagens internacionais recentes. No dia 11 de março de 2020, foi confirmado o primeiro caso de transmissão local, também no HIAE. Uma proporção relevante de todos os pacientes com infecção confirmada por COVID-19 havia sido diagnosticada no HIAE no momento da análise.

Os pacientes de nossa série tinham média de idade de 39,9 anos e eram na maioria homens (56,9%). Os estudos que descrevem as características demográficas na população geral infectada mostram idade mediana de 47 anos,^( 7 , 12 )^e a proporção de homens foi de 58,1% no relatório chinês^( 7 )^e de 50% no de Cingapura.^( 12 )^Os sintomas respiratórios foram semelhantes aos dos pacientes descritos em relatórios da China, Estados Unidos e Europa.^( 7 , 9 , 13 )^No entanto, a média de dias de sintomas foi muito menor em nossa série (2,8 dias *versus* 13 dias em Cingapura,^( 12 )^7 dias nos Estados Unidos^( 13 )^e 7 dias na China.^( 3 )^

Embora tenha sido relatada pela maioria dos pacientes, a febre esteve presente em apenas 15,6% dos pacientes no momento da avaliação inicial no hospital, sugerindo que, talvez, ela não tenha que ser levada em conta para determinar a gravidade da doença, além de os algoritmos diagnósticos que utilizam a febre como parte do exame poderem mascarar o número total de casos e atrasar o diagnóstico. A prevalência de doenças crônicas foi muito maior no grupo de pacientes hospitalizados (50%) em relação ao dos não hospitalizados (15,2%). Essa prevalência foi ainda maior no subgrupo admitido na UTI (80%).

A média de idade dos pacientes hospitalizados foi superior à dos não hospitalizados (51,8 *versus* 38,6 anos) e a necessidade de hospitalização aumentou com a idade (7,8% para pacientes entre 12 e 49 anos, 33,8% entre 50 e 64 anos e 45,5% para maiores de 65 anos). Nessa série de casos brasileiros, a internação foi necessária em 72 (14,1%) pacientes, e 20 deles demandaram cuidados intensivos, representando 27,8% do total de internações – um número muito superior ao da série chinesa, na qual apenas 5% necessitaram de UTI.^( 7 )^

A maioria dos pacientes foi internada em UTI devido à insuficiência respiratória hipoxêmica aguda, que necessitava de suporte ventilatório. A ventilação mecânica invasiva foi necessária em 65% dos pacientes da UTI (18,1% do total de internações), ao passo que 20% foram tratados com ventilação mecânica não invasiva. A necessidade de ventilação mecânica invasiva foi semelhante à de uma série de pacientes internados em UTI relatada nos Estados Unidos (75% registrados no estado de Washington)^( 13 )^e menor do que a relatada em publicação italiana (88% na Lombardia),^( 9 )^porém superior à dos relatórios chineses (47%, 42% e 30% relatados em Wuhan; metade deles tratados com oxigenação por membrana extracorpórea).^( 3 , 14 , 15 )^Levando em consideração o uso de ventilação não invasiva, o índice foi novamente semelhante ao relatado em Washington (19%)^( 12 )^e inferior aos da China (42%, 56% e 62%, em Wuhan, incluindo os pacientes que necessitaram de cânula nasal de alto fluxo).^( 3 , 14 , 15 )^Três pacientes (15% dos pacientes internados na UTI) apresentaram lesão renal aguda com necessidade de terapia renal substitutiva contínua. Entre estes, apenas um apresentava doença renal crônica. A prevalência de doença renal crônica foi de 2,9% entre os pacientes hospitalizados segundo relatório chinês^( 14 )^e de 21% entre os pacientes admitidos na UTI na série norte-americana (21%).^( 13 )^

Este estudo tem limitações importantes. Primeiro, parte dos casos tinha informações incompletas documentadas nos prontuários, e a documentação da história clínica dos pacientes não era homogênea entre todos eles. Essa é uma limitação comum em estudos observacionais retrospectivos, levando-se em consideração que a geração de dados foi orientada em termos clínicos e não de forma sistemática. Segundo, como muitos pacientes permaneceram no hospital e os desfechos eram desconhecidos no momento do encerramento da coleta de dados, censuramos os dados referentes aos desfechos clínicos no momento da análise. Terceiro apenas os pacientes internados no HIAE foram incluídos no grupo hospitalizado, e não há documentação das internações realizadas fora da nossa rede. Por fim, este estudo incluiu apenas pacientes atendidos ambulatorialmente ou internados no HIAE, não tendo sido levados em consideração os casos assintomáticos e leves que não procuraram assistência médica. Assim, nossa coorte de estudo pode representar casos mais graves de COVID-19.

## CONCLUSÃO

Até o momento, não há estudos no Brasil relatando as características dos pacientes com diagnóstico de COVID-19. O Brasil é o país do hemisfério sul com o maior número de casos confirmados da doença, e o Hospital Israelita Albert Einstein é o centro onde o primeiro paciente foi diagnosticado, com amostra representativa de todos os casos confirmados de COVID-19 no país. Os resultados apresentados neste estudo podem ser relevantes para o Brasil e para outros países com características semelhantes, que começaram a lidar com essa pandemia.
